# Beyond Antibiotics: The Expanding Role of Non-Antibiotic Therapies in Veterinary Ophthalmology

**DOI:** 10.3390/vetsci13050461

**Published:** 2026-05-09

**Authors:** Marta Leiva, Rita Vilao Cardoso, Laura Gaztelu, Teresa Peña

**Affiliations:** 1 Servei d’Oftalmologia, Fundació Hospital Clínic Veterinari, Carrer del Hospital s/n, Universitat Autònoma de Barcelona, 08193 Bellaterra, Barcelona, Spain; rita.vilao@uab.cat (R.V.C.); laura.gaztelu@uab.cat (L.G.); teresa.pena@uab.cat (T.P.); 2 Departament de Medicina i Cirurgia Animals, Facultat de Veterinària, Travessera dels Turons s/n, Universitat Autònoma de Barcelona, 08193 Bellaterra, Barcelona, Spain

**Keywords:** infection, antibiotic, resistance, ocular surface, eye

## Abstract

The sustained increase in bacterial resistance has driven the search for therapeutic alternatives that may help reduce antibiotic use, particularly in empirical treatments. In this context, topical ophthalmic antiseptics have emerged as effective and safe tools, suitable for both perioperative prophylaxis and the management of mild or superficial ocular diseases. Their broad spectrum of activity—encompassing bacteria, fungi, viruses, and protozoa—together with the low likelihood of inducing resistance, establishes them as valuable partners in daily clinical practice. This article reviews the current evidence on the use of antiseptics in veterinary ophthalmology, focusing on their clinical applications, therapeutic benefits, and limitations. It also highlights the need for further studies to establish species-specific, disease-specific, and context-based protocols.

## Simple Summary

The steady growth of bacterial resistance has encouraged the development of non-antibiotic therapeutic options to limit antibiotic dependence. Among these, topical ocular antiseptics have shown considerable promise as effective and safe agents for both preventive and therapeutic ophthalmic use. This study aimed to review the current literature on the use of antiseptics in veterinary ophthalmology, describing the main agents employed and analyzing their respective advantages and limitations. The study concludes that several antiseptic agents are available for clinical use in veterinary ophthalmology, and they can be effectively applied in cases of mild to moderate ocular disease severity. The study holds societal relevance by promoting rational therapeutic practices that can help decrease antibiotic dependence and, consequently, slow the development of antimicrobial resistance—an issue with implications for both animal and human health.

## 1. Introduction

Antibiotic resistance is one of the major current challenges in both human and veterinary medicine. In ophthalmology, the empirical or indiscriminate use of topical antibiotics has contributed to the emergence of resistant strains such as *Staphylococcus pseudintermedius* and *Pseudomonas aeruginosa*, characterized by complex mechanisms of therapeutic evasion [1,2]. These resistances not only compromise treatment efficacy but may also lead to serious clinical consequences, including therapeutic failure and, in advanced cases, irreversible vision loss [3,4]. Additionally, excessive reliance on antibiotics has likely favored biofilm formation, whose heightened resistance to antimicrobial agents represents a significant obstacle to effective treatment.

To counteract this trend, several strategies have been proposed to promote the rational use of antimicrobials. These include: (1) restricting empirical antibiotic use, (2) encouraging treatments guided by microbial culture or ocular cytology, (3) the use of antibiotic-potentiating agents, and (4) the incorporation of topical antiseptics when clinically feasible. Additionally, (5) antimicrobial stewardship programs aimed at monitoring and regulating antibiotic use are promoted [5]. In this context, ophthalmic antiseptics are emerging as a valuable complementary therapeutic tool. Unlike antibiotics, they act on multiple microbial structures through nonspecific mechanisms, significantly reducing the likelihood of resistance [6,7]. They also exhibit a broad spectrum of activity against Gram-positive and Gram-negative bacteria, fungi, viruses, certain protozoa and biofilms, making them highly versatile agents in diverse clinical scenarios [8]. Notably, bacterial biofilms are more resistant to both the immune system and antibiotic agents than their free-living planktonic counterparts; thus, infections related to biofilms are persistent, frequently recurrent and may lead to irreversible ocular damage.

In addition to the active compound, the quality and characteristics of ophthalmic formulations play a critical role in determining the safety and efficacy of non-antibiotic therapies. Parameters such as pH, osmolarity, buffering capacity, and excipient composition can significantly influence ocular surface tolerance, drug stability, and bioavailability [9,10]. In particular, pH is a key factor, as deviations from the physiological tear pH may lead to ocular irritation, increased blinking, and reduced contact time, ultimately affecting therapeutic effectiveness [9]. Furthermore, formulation-related factors such as viscosity, preservative systems, and the presence of surfactants or chelating agents may modulate drug penetration and antimicrobial activity, especially in the context of biofilm-associated infections [11]. Therefore, the selection of well-designed ophthalmic formulations is essential to optimize clinical outcomes and minimize adverse effects.

The aim of this article is to critically review the role of non-antibiotic therapies in veterinary ophthalmology, with a primary focus on dogs and cats. We assess their efficacy, safety, and current clinical applications, as well as their potential to reduce reliance on topical antibiotics and improve the management of biofilm-associated infections. In addition, we provide practical recommendations for their rational and integrated use in clinical practice and identify key areas for future research.

This narrative review was based on a targeted literature search performed in PubMed, Web of Science, and Scopus using keywords related to “antiseptics”, “biocides”, “veterinary ophthalmology”, and “ocular surface diseases”. The search primarily focused on studies published between January 2000 and February 2026, with emphasis on recent literature. However, earlier landmark studies were included when relevant to support key concepts. Both veterinary and human ophthalmology studies were considered, and articles were selected based on their scientific relevance and clinical applicability.

## 2. Main Applications of Antiseptics in Veterinary Ophthalmology

The use of antiseptics is increasingly recognized as a valuable strategy for the management of mild ocular infectious diseases, as well as an adjunctive therapy in chronic or recurrent infections. However, although several antiseptics have shown clinical efficacy in the treatment or supportive management of specific ophthalmic conditions, the overall body of evidence remains limited. Consequently, prospective studies and controlled clinical trials are required to validate their therapeutic effectiveness in active ocular infections.

From our perspective, the use of ocular antiseptics is particularly relevant in four main settings: (1) perioperative antisepsis, (2) routine ocular surface hygiene, (3) management of mild ocular surface and adnexal disorders (e.g., tear film abnormalities, conjunctivitis, blepharitis, simple corneal ulcers), and (4) treatment of recurrent or chronic keratitis or keratoconjunctivitis. In our experience, ocular surface cytology is a rapid and effective diagnostic tool that supports an evidence-based selection of the initial therapeutic approach and helps determine whether the use of antiseptics, antibiotics, or a combination of both is most appropriate.

When no bacterial forms are observed cytologically, or when only scarce extracellular organisms are detected, the findings may correspond to normal flora or contamination [12], and therefore we opt for the exclusive use of antiseptics. In contrast, the presence of intracellular bacteria generally warrants the initiation of antibiotic therapy [13]. Nevertheless, if such intracellular forms are few in number, the use of antiseptics may be considered, reserving antibiotics for situations with a moderate to significant bacterial load. Finally, in chronic or recurrent lesions, in addition to performing bacterial culture, we may combine some antiseptics with antibiotic treatment to enhance the penetration of the latter and optimize the clinical response [14].

For the purposes of this review, the term mild to moderate ocular disease refers to superficial, non-vision-threatening conditions with limited clinical severity, including mild to moderate conjunctival hyperemia or discharge, superficial corneal epithelial involvement without stromal loss, absence of stromal melting, no marked intraocular inflammation, no severe pain, and no immediate risk of perforation or permanent vision loss. Typical examples include uncomplicated conjunctivitis, mild blepharitis, tear film instability, superficial keratitis, and uncomplicated superficial corneal ulcers. In contrast, deep corneal ulcers, stromal malacia, severe keratitis, marked uveitis, progressive infection, or vision-threatening disease should not be considered within this category and generally require more intensive diagnostic and therapeutic management.

## 3. Main Antiseptics in Veterinary Ophthalmology

In both human and veterinary ophthalmology, various types of antiseptics have been used for prophylactic and therapeutic purposes. We describe below the main antiseptic agents used in the ophthalmic field, along with their mechanisms of action and key characteristics. Table S1 provides a practical comparative summary of the most relevant information on the antiseptics most used, including their mechanisms of action, antimicrobial spectrum, and clinical features. It also includes a list of veterinary products marketed in Europe and the USA, as well as selected human products that may be used off-label.

### 3.1. Povidone–Iodine

One of the most widely used antiseptics is povidone–iodine, a complex of molecular iodine with a carrier polymer that acts by slowly and consistently releasing free iodine. This iodine penetrates microbial cell membranes and binds to proteins, lipids and nucleic acids, leading to their denaturation. It has a very broad antimicrobial spectrum, including Gram-positive and Gram-negative bacteria, fungi, viruses, protozoa, spores and biofilms [15]. When used in diluted concentrations (0.2–1%), it has been shown to be effective for preoperative antisepsis and well tolerated by the ocular surface [16], inhibiting biofilm formation and reducing the stability of established biofilms [17]. In addition, a recent in vitro study proved this compound to be efficient against bacteria associated with equine infectious keratitis at concentrations as low as 0.00005–0.0256% [18]. However, it may cause irritation if applied undiluted or if contact with the ocular surface is prolonged.

In veterinary ophthalmology, povidone–iodine–based formulations are frequently used off-label as safe and effective alternatives when ocular antisepsis is required [19,20], as well as in the management of conditions such as blepharitis, conjunctivitis, and superficial keratitis. As summarized in Table S1, there are currently no commercially available veterinary-specific ophthalmic products containing povidone–iodine in Europe or the United States. An exception is found in India, where a veterinary formulation (Visiotears^®^, Sava Vet, Wadhwan City, Surendranagar, Gujarat, India) is available.

### 3.2. Polyhexanide

Polyhexanide is a broad-spectrum antiseptic that has gained increasing attention in ophthalmology due to its excellent safety profile and proven efficacy against Gram-positive and Gram-negative bacteria, fungi, protozoa and biofilms [20]. Its mechanism of action involves interaction with microbial cell membrane phospholipids, resulting in membrane disruption and subsequent cell death. In veterinary ophthalmology, polyhexanide has been incorporated into commercial formulations at very low concentrations (e.g., 0.0001%), in combination with other antiseptics, as part of the topical management of spontaneous chronic corneal epithelial defects (SCCEDs) [21]. In human medicine, it has been successfully used against *Acanthamoeba* spp. [22], although in veterinary medicine its use in this context is based primarily on extrapolation from that evidence.

Polyhexanide-containing ophthalmic formulations are commercially available in Europe; however, as outlined in Table S1, most are developed for human use rather than veterinary application. In contrast, veterinary-specific options are scarce, although Septostil^®^ (Vetilea S.L, Barcelona, Spain) is commercially available in Europe, combining 0.0001% polyhexanide with other antiseptic compounds.

### 3.3. Hypochlorous Acid (HOCl)

Over the past few years, hypochlorous acid at low concentrations has emerged as a valuable antiseptic, recognized for its favorable safety profile and its broad antimicrobial spectrum, encompassing bacteria, fungi, viruses and biofilms [15,23]. This compound is naturally produced by neutrophils as part of the innate immune response and acts by inducing oxidative injury to microbial structures. Its high efficacy, combined with minimal toxicity, makes it a promising option for certain ophthalmic indications, provided it is used at concentrations of 0.01% [24,25]. In ophthalmology, its applications include eyelid hygiene in cases of blepharitis, management of conjunctivitis and superficial keratitis, and perioperative prophylaxis [24,25]. Although it does not replace antibiotics in severe infections, it represents a safe and effective alternative for reducing the use of topical antimicrobials and for treating ocular surface biofilms. It is worth noting that it is suitable for long-term use [24].

Hypochlorous acid-based ophthalmic formulations are currently available for veterinary use in both Europe and the United States, as summarized in Table S1. In Europe, Hypochlorine Eye Care^®^ (JTPharma, Boadilla del Monte, Madrid, Spain) represents a veterinary-specific product containing hypochlorous acid at the recommended concentration. In the United States, several veterinary formulations are also commercially available, including Vetericyn Plus^®^ Antimicrobial Ophthalmic Gel (Innovacyn Inc., Rio alto, California, United States of America (USA)), MicrocynAH^®^ Ophthalmic Gel (Compana Pet Brands, Chesterfield, MO, USA), and HICC Pet^®^ Gentle Antimicrobial Eye Rinse for Dogs (Bellevue, WA, USA) These products provide species-specific options for ocular surface antisepsis and periocular hygiene in clinical practice.

### 3.4. Ethylenediaminetetraacetic Acid (EDTA)

EDTA is a chelating agent widely used in ophthalmology due to its ability to bind divalent cations such as calcium and zinc [26]. In veterinary medicine, EDTA has long been employed as an adjunctive treatment in complicated corneal ulcers, where it acts as an antiproteolytic agent at concentrations of 0.1–1% [27], and in calcific keratopathy, where it is used as a calcium chelator at 1–5% [28].

Although the antiseptic properties of EDTA have been recognized for more than 50 years [29], its application as an antiseptic and antibiotic adjuvant at concentrations of 0.05–0.1% is relatively recent. Its main value resides in its role as a therapeutic enhancer, potentiating and synergizing the activity of antibiotics [30]. Its mechanism of action involves destabilization of the bacterial outer membrane, thereby facilitating antibiotic penetration [31]. This effect is relevant against both Gram-positive and Gram-negative bacteria, but is particularly important in the latter, whose double membrane acts as a physical and functional barrier that limits the action of many antimicrobials [32]. Consequently, in chronic or persistent infections caused by Gram-negative bacteria such as *Pseudomonas* spp., *Chlamydia* spp., *Enterobacter* spp., *Bordetella bronchiseptica* or *Pasteurella multocida*, EDTA helps overcome this barrier and enhances antibiotic efficacy. Accordingly, it has recently been incorporated into ophthalmic formulations together with surfactants (such as polysorbate 80) or buffering agents (such as tromethamine) to potentiate the efficacy of topical antimicrobials in refractory or chronic conditions [30]. Although further clinical evidence is needed to establish standardized protocols, current experience supports the use of EDTA as a valuable adjunct in the management of refractory ocular infections.

As summarized in Table S1, several human ophthalmic formulations are available for periocular hygiene; however, veterinary-specific products are also commercially available in both Europe and the United States. In Europe, Optican Limpiador de Ojos^®^ (Cantabria lab Stangest, Valls, Tarragona, Spain) is indicated for routine periocular cleansing and tear stain management, while in the United States, I-LID ’N LASH VET^®^ (I-MED Animal Health, Saint-Laurent, Quebec, Canada) is specifically formulated for eyelid hygiene and the management of blepharitis in companion animals.

**Tromethamine (tris),** also known as tris (hydroxymethyl)aminomethane, is a buffering agent incorporated into ophthalmic formulations for therapeutic purposes, particularly due to its ability to modulate pH and enhance the activity of other compounds. Although Tris does not have intrinsic antiseptic properties, it is frequently combined with agents such as EDTA and surfactants to promote disruption of the bacterial outer membrane, reducing inherent resistance and enhancing the penetration of topical antibiotics [31]. This synergistic interaction is especially valuable in biofilm-associated ocular infections, where bacteria assume a more treatment-resistant phenotype [32]. In veterinary medicine, Tris–EDTA solutions may be used as adjuvants for cleansing and preparing the ocular surface prior to antimicrobial application, particularly in chronic or refractory cases. Tris has demonstrated good tolerance by the ocular surface and represents a useful component in combined therapies to optimize treatment efficacy without increasing local toxicity.

In veterinary ophthalmology, products containing both Tris and EDTA such as ASTER^®^ Trisoftal Wipes (VetNova, Madrid, Spain) are available in Europe while TrisOphtho^®^ Eye Wipes (DermaZoo™ Pharma, Potomac, Mayland, USA) are available in the United States (Table S1).

**Polysorbate 80**, also known as Tween 80, is a nonionic surfactant widely used in ophthalmic pharmaceutical formulations due to its ability to solubilize lipophilic compounds and stabilize emulsions. Although it is not an antiseptic in the strict sense, it has been reported to reduce microbial load, primarily by altering bacterial cell membrane integrity and facilitating biofilm dispersion [33]. In veterinary ophthalmology, polysorbate 80 is mainly used as an excipient in eye drops and ophthalmic solutions and, in some cases, as an adjuvant in combination with antimicrobials, enhancing their penetration and bioavailability. Although most of the available evidence derives from studies in human ophthalmology [34], this information is extrapolated to the veterinary field, where its use is considered particularly relevant in the management of chronic or recurrent ocular infections. In addition, its ocular safety profile is well established, showing good tolerance on the ocular surface [34].

In veterinary practice, a commercial formulation combining Tris, EDTA and polysorbate 80 is currently available (Ophtaprime^®^, Domes Pharma, Pont-du-Château, France), which makes it an appealing option for use in small animals [35]. In addition to acting as an ocular cleansing solution, it prepares the ocular surface and enhances antibiotic activity. However, due to the surfactant effect of polysorbate 80, its use is generally limited to a maximum of 15 days. In patients with tear film disturbances, regular use of this combination may help reduce the proliferation of pathogenic microorganisms and maintain a controlled microbial load, thereby decreasing recurrence rates, improving ocular comfort, and limiting repeated antibiotic use. Similar combinations are commercially available in human ophthalmology in both Europe and the United States (see Table S1). In veterinary medicine, a comparable formulation is also available, such as Optixcare Eye Care^®^ (CLC Medica LLC, ON, Canada), which combines polysorbate 80 with sodium citrate and disodium EDTA.

**Sodium citrate**, known primarily for its role as a mild chelating agent, may exert a synergistic effect when used as an adjuvant in combination with antibiotics or other antiseptics, such as EDTA or Tris-buffered formulations [36]. Although it is not as potent as EDTA, it is considered safer for chronic and daily administration [37]. Its mechanism of action is based on its ability to sequester divalent cations—primarily calcium—which are essential for the integrity of the bacterial outer membrane [38]. Similar to EDTA, it destabilizes the bacterial cell wall, thereby facilitating the penetration of other antimicrobial agents.

In veterinary ophthalmology, sodium citrate is available in Pet Hydra Drops^®^ (Meraky by KF Srl, Luxembourg) in Europe. In human ophthalmology, sodium citrate is mainly used in artificial tears and lubricating emulsions with antioxidant and modulatory properties—such as Hyabak^®^ (Laboratoires Théa, Clermont-Ferrand, France), Emustil^®^ (SIFI, Sicilia, Italy), Optive^®^ Fusion and Refresh (Allergan, Dublin, Ireland) and Vismed^®^ (Brudylab, Barcelona, Spain) in Europe, Cationorm^®^ (Santen, Osaka, Japan in Asia and Systane^®^ Hydration and Balance (Alcon, Fort Worth, TX, USA) and in the United States—where it acts as an adjunct in protecting the ocular surface and stabilizing the tear film.

### 3.5. Boric Acid

Boric acid is a traditional compound with mild antimicrobial properties, commonly used in ophthalmic solutions as an adjuvant or buffering vehicle. Its mechanism of action involves interference with microbial enzymatic metabolism, thereby limiting bacterial and fungal growth [39]. Although its antiseptic effect is weaker than that of other agents, its good ocular tolerance and soothing properties make it suitable for formulations used in mild conjunctivitis and routine ocular surface hygiene [37].

In veterinary medicine, some commercial products contain boric acid as a key ingredient (Ocryl^®^, Domes Pharma, Pont-du-Château, France; Siccostil Protect^®^, Vetilea, Barcelona, Spain; Lavatears^®^, Santgar SA, Ciudad de Mexico, Mexico; Angels’ Eyes^®^, H&C Animal Health, Colorado, USA). These formulations make use of the mild antimicrobial effect of boric acid for ocular hygiene, relief of mild irritations, and post-diagnostic rinsing. This compound is also incorporated into solutions aimed at managing tear staining syndrome. Its gentle antimicrobial activity [37] and its ability to modulate the periocular microenvironment—particularly through pH adjustment and reduction of the superficial bacterial load—may contribute to limiting early microbial adhesion and biofilm formation on the ocular surface, although direct evidence supporting a significant antibiofilm effect is currently limited. In human ophthalmology, boric acid is commonly included as a component of over-the-counter ocular formulations in both Europe and the United States (see Table S1).

### 3.6. Hexamidine

Hexamidine, in its salt form (hexamidine diisethionate), is an antiseptic with broad-spectrum antimicrobial activity, particularly effective against Gram-positive bacteria, some Gram-negative bacteria and certain protozoa such as *Acanthamoeba* spp. [8,40]. It exerts its effect by disrupting the microbial cell membrane, leading to cell lysis [37]. This mechanism may also contribute to interference with early microbial adhesion and biofilm formation, although direct evidence of a significant antibiofilm effect remains limited. Hexamidine demonstrates good ocular tolerance when used at appropriate concentrations; however, it may cause irritation if applied in highly concentrated solutions or over prolonged periods. In veterinary ophthalmology, there are no specific studies supporting its use as an independent active ingredient; however, its application is based on extrapolation from human evidence and on its inclusion within combined commercial formulations [8,40,41].

In veterinary practice, a commercial formulation is available (Septostil^®^, Vetilea, Barcelona, Spain), containing hexamidine (0.05%), polyhexanide (0.0001%), EDTA (0.05%) and phosphates, providing an interesting profile for the treatment of mild ocular surface infections. In such combinations, the potential antibiofilm activity is likely driven primarily by agents such as polyhexanide and EDTA, which have demonstrated more consistent efficacy against biofilm-associated microorganisms.

### 3.7. Ozone

Ozone has emerged as a promising agent in veterinary ocular antisepsis due to its strong oxidative potential [41]. In addition, it exhibits anti-inflammatory and wound-healing properties, further supporting its use as an adjuvant. In human medicine, low concentrations have been reported (<5 ppm in aqueous solutions, <20 µg/mL in eye drops) to minimize topical irritation [42], although no standardized guidelines currently define optimal dosing. In veterinary medicine, no standardized concentrations have been established, and values are generally extrapolated from human protocols. In small animals, ozone is mainly administered as ozonated water for ocular surface cleansing and disinfection—particularly in cases of corneal ulcers or infectious keratitis—and as ozonated oils applied periocularly to promote wound healing and control infection [41,43]. However, clinical application requires caution, as high concentrations or improper formulations (non-buffered water or non-stabilized ozonated agents) may cause chemical keratitis [44]. Furthermore, repeated or uncontrolled exposure may induce chronic oxidative stress in epithelial and endothelial cells, impairing healing.

Regarding biofilm-associated infections, ozone has demonstrated the ability to disrupt microbial biofilms and reduce biofilm biomass in in vitro studies, primarily through oxidative damage to extracellular polymeric substances and microbial cell membranes [45,46]. However, the clinical relevance of these findings in veterinary ophthalmology remains unclear, as in vivo evidence is still limited and no standardized protocols have been established.

Despite encouraging preliminary results [47], scientific evidence in veterinary ophthalmology remains limited, and further controlled studies are needed to establish safe and effective treatment protocols. To the authors’ knowledge, no veterinary-licensed ophthalmic ozone products are currently available, and its use relies on off-label formulations derived from human medicine, such as Ozonest^®^ (Laboratorios Esteve, Barcelona, Spain). Since the bactericidal effect of ozone has been shown to be less potent and slower in onset than 0.6% povidone–iodine, its perioperative application is not justified [7].

### 3.8. Biosecur^®^

Biosecur^®^ is a patented plant-derived extract obtained from citrus (*Citrus aurantium*), rich in bioflavonoids and polyphenols, with antimicrobial activity against bacteria, fungi and viruses [48]. It has been incorporated into human ophthalmic formulations such as Oftasecur^®^ (Offhealth, Firenze, Italy), demonstrating in vitro efficacy against *Candida albicans*, *C. auris* and even biofilms, according to available experimental studies and technical data [49]. However, current evidence supporting a significant antibiofilm effect is limited and largely restricted to in vitro or manufacturer-reported data, with a lack of independent studies confirming its efficacy in clinically relevant conditions. To date, no veterinary-approved ophthalmic products containing Biosecur^®^ are available. Therefore, any use in animals would constitute off-label administration and should be preceded by a critical evaluation of its safety and efficacy.

Although it does not replace broad-spectrum antiseptics such as polyhexanide or povidone–iodine, Biosecur^®^ represents a useful option for ocular hygiene and is also marketed in wipe form for eyelid and conjunctival cleansing [50]. Its favorable tolerance allows for repeated use; however, its antimicrobial effect is limited compared with conventional ophthalmic antiseptics [8], and its role in the prevention or management of biofilm-associated infections remains to be established.

### 3.9. Ultraviolet (UV) Radiation

Ultraviolet (UV) radiation—particularly UV-C (wavelengths between 200 and 280 nm)—has been shown to be effective against bacteria, viruses and fungi. In veterinary ophthalmology, its use as an ocular antiseptic remains limited and experimental; however, it is gaining interest as a non-pharmacological tool for the management of resistant corneal infections [50]. Photodynamic therapy with UV-C, either alone or in combination with riboflavin (corneal cross-linking), has been used in cases of infectious keratitis in animals, particularly in horses and dogs, with the aim of reinforcing corneal structure and reducing microbial load [50,51]. In addition to its antimicrobial effect, this treatment can induce the formation of covalent cross-links within corneal collagen, contributing to stabilization of progressive ulcers.

Regarding biofilm-associated infections, UV-C irradiation and photoactivated cross-linking have demonstrated the ability to reduce microbial load and disrupt biofilm structure in experimental studies, primarily through direct DNA damage and the generation of reactive oxygen species [52,53]. However, the penetration of UV light is limited, and its efficacy against mature or deep biofilms may be reduced. Moreover, clinical evidence supporting its antibiofilm activity in veterinary ophthalmology remains scarce, and standardized treatment protocols have not yet been established.

Nevertheless, its application must be performed under strictly controlled conditions, as inappropriate UV exposure can result in tissue damage and adverse effects on epithelial and endothelial cells. Despite its potential, further clinical studies in veterinary medicine are required to define its safety, efficacy and possible indications in routine practice.

Beyond the antiseptics, several new agents are being investigated for potential application in veterinary ophthalmology. N-acetylcysteine has demonstrated significant in vitro activity against major pathogens responsible for infectious keratitis in dogs and cats, supporting its potential role as an ocular antiseptic [8,54]. However, in vivo studies are still needed to confirm its safety, clinical efficacy and dosage protocols in companion animals. Similarly, a recent study found that 0.1% polyquaternium-133 exhibits notable antiseptic activity against common ocular pathogens, even at low concentrations, suggesting a promising profile for future ophthalmic formulations [8].

## 4. Antiseptics with Limited Use in Veterinary Ophthalmology

Not all available antiseptic agents are suitable for ocular application, as some can induce toxicity to ocular structures or lack sufficient support in veterinary medicine. The antiseptics described below require restricted use or careful clinical consideration in veterinary ophthalmology.

### 4.1. Chlorhexidine

Chlorhexidine is one of the most widely used compounds for general disinfection. It is a cationic biguanide that exerts its antimicrobial effect by disrupting the bacterial cell membrane. At low concentrations, it acts as a bacteriostatic agent, whereas at higher concentrations it becomes bactericidal. Although its spectrum of activity primarily includes Gram-positive bacteria, it also shows some efficacy against Gram-negative bacteria, fungi and viruses. However, its ocular application requires caution, as concentrations above 0.05% may cause significant irritation to the cornea and conjunctiva [55]. For this reason, it is considered safe only in formulations specifically designed for ophthalmic use, such as certain chlorhexidine digluconate solutions ≤0.05% as the Iryplus^®^ ocular cleanser (Fatro, Barcelona, Spain) and DROPSEPT^®^ (Servimed Industrial, Rome, Italy).

### 4.2. Hydrogen Peroxide (H_2_O_2_)

Despite its well-known antiseptic potency and ability to eliminate bacteria, viruses, fungi and spores through free radical formation, hydrogen peroxide is highly toxic to ocular tissues. Due to its cytotoxic effects on the cornea and conjunctiva, its direct use on the ocular surface is entirely contraindicated [56,57]. Its application is restricted to instrument disinfection or contact lens cleaning systems, and only when followed by a neutralization step.

### 4.3. Tea Tree Oil (Melaleuca alternifolia)

Tea tree oil possesses recognized antimicrobial, antifungal and anti-inflammatory properties [58]. However, its direct ocular application in veterinary ophthalmology is very limited due to its irritant and cytotoxic effects on ocular tissues, particularly in concentrated forms. Diluted or compounded formulations have been used for the treatment of blepharitis, palpebral demodicosis and other periocular conditions, mainly in dogs. Its efficacy against *Demodex* spp., Gram-positive bacteria and some fungi make it an interesting option for periocular antisepsis [59]. Nonetheless, its use requires extreme caution to avoid direct contact with the ocular surface, which can be challenging in clinical practice.

### 4.4. Silver Compounds

Silver-based compounds, such as silver nitrate and silver nanoparticles, have demonstrated notable activity against multidrug-resistant microorganisms [60], leading to renewed interest in their medical use. However, their application in veterinary ophthalmology remains very limited and is largely confined to experimental settings. The high risk of ocular toxicity [37], limited clinical experience in animals and the absence of formulations specifically designed for ophthalmic use currently prevent their routine clinical application.

In summary, these considerations highlight the importance of carefully evaluating the safety and efficacy profile of each antiseptic prior to ocular use, always prioritizing agents with favorable clinical evidence and a low risk of toxicity.

## 5. Advantages and Limitations of Ocular Antiseptics

One of the main advantages of antiseptics is undoubtedly their (1) broad-spectrum activity [37]. This “multitarget” effect is due to nonspecific mechanisms of action—such as oxidation or microbial protein denaturation—which also means that (2) the likelihood of resistance development is extremely low or virtually negligible, even with repeated use. Another notable advantage is (3) their rapid onset of action and ability to significantly reduce microbial load without the need to await culture results [61]. This makes them ideal for use in the early phases of treatment, perioperative procedures or in patients with mild infections or unconfirmed etiologies. In addition, many antiseptics are (4) low-cost and (5) stable, which facilitates routine use both in clinical settings and at home. Furthermore, (6) some of these compounds enhance the penetration of antibiotics and other antiseptics [37] thereby potentiating clinical outcomes. This is particularly relevant in chronic or recurrent infections, where the combination with chelating or potentiating agents (such as EDTA) improves the penetration of antibiotics into Gram-negative bacteria, which are traditionally more refractory to treatment due to their double membrane and tendency to form biofilms.

Despite their advantages, ocular antiseptics also present several limitations that must be considered in veterinary practice. Firstly, (1) clinical evidence in animals remains scarce for many agents, particularly regarding controlled studies, ocular pharmacokinetics and species-specific protocol validation. Although extrapolating data from human medicine can be useful, it is not always appropriate due to anatomical and physiological differences among species. Another important consideration is (2) the potential irritative effect of certain antiseptics when used at inappropriate concentrations or in formulations not designed for ophthalmic use. Moreover, (3) their activity is not always selective for pathogens; prolonged or inappropriate use may delay healing or damage healthy tissues, underscoring the need for adherence to product guidelines. It should also be noted that many of these compounds (4) have poor penetration and thus are limited to external ocular surfaces (conjunctiva, eyelids, superficial cornea) and are ineffective in intraocular infections or deep keratitis. Although antiseptics play a valuable role in reducing antibiotic use, (5) they should not be considered universal substitutes. In severe, progressive infections or deep corneal ulcers at risk of perforation, culture-based antibiotic therapy and sensitivity testing remain the clear standard of care. Finally, (6) interspecies variability represents an important limitation of non-antibiotic therapies. Different animal species exhibit relevant anatomical and physiological variations of the ocular surface that may influence the safety, tolerability, and efficacy of these treatments. Factors such as tear film composition and stability, corneal structure, epithelial permeability, and baseline ocular surface microbiota differ significantly across species, which may directly affect drug distribution, pharmacokinetics, and therapeutic response [62,63,64,65]. These differences are particularly relevant for non-antibiotic agents, as their mechanisms of action, concentration-dependent effects, and potential epithelial toxicity may not be directly extrapolated between species. Therefore, a species-adapted approach should be considered when selecting and formulating ophthalmic treatments in veterinary patients.

Although antiseptics are generally associated with a low risk of resistance development due to their multitarget mechanisms of action, emerging evidence suggests that microorganisms may exhibit adaptive responses following repeated or sublethal exposure. These adaptations may include changes in membrane permeability, upregulation of efflux pumps, or biofilm-related tolerance, which in some cases have been associated with reduced susceptibility not only to antiseptics but also to certain antibiotics [66,67]. While the clinical relevance of these findings in veterinary ophthalmology remains unclear, they highlight the importance of appropriate concentration, formulation, and duration of use. Therefore, antiseptics should be applied judiciously and as part of an integrated therapeutic approach, rather than as indiscriminate or prolonged treatments.

## 6. Conclusions

In conclusion, biocides and other non-antibiotic therapies represent valuable tools in veterinary ophthalmology, particularly for reducing reliance on topical antibiotics and supporting antimicrobial stewardship. Their broad-spectrum activity, rapid onset of action, and low propensity for resistance make them especially suitable for perioperative antisepsis, routine ocular hygiene, and the management of mild to moderate ocular surface diseases.

From a clinical perspective, several key recommendations can be derived. First, the use of non-antibiotic therapies should be guided by clinical examination and, whenever possible, supported by diagnostic tools such as ocular cytology to differentiate between colonization and infection. Second, antiseptics may be considered as first-line treatment in mild or superficial conditions, while reserving antibiotics for cases with clear evidence of intracellular infection, moderate to severe bacterial load, or deep corneal involvement. Third, in chronic or recurrent infections, the combined use of antiseptics with antibiotics or adjuvant agents (e.g., EDTA-based formulations) may enhance therapeutic efficacy and improve drug penetration. Fourth, the selection of formulations specifically designed for ophthalmic use is essential to minimize toxicity and ensure ocular surface compatibility.

Importantly, these therapies should not be regarded as universal substitutes for antibiotics. In severe, progressive infections or in cases at risk of corneal perforation, culture-based antibiotic therapy remains the standard of care. In addition, interspecies variability, formulation characteristics, and appropriate dosing regimens must be carefully considered to optimize safety and efficacy.

Despite promising clinical applications, significant knowledge gaps remain. Future research should focus on controlled clinical trials in veterinary species, pharmacokinetic and pharmacodynamic studies, and the development of standardized, species-specific protocols. Further investigation into the role of non-antibiotic therapies in biofilm-associated infections and their interaction with conventional antimicrobials is also warranted.

Overall, when used appropriately and within an evidence-based framework, non-antibiotic therapies constitute a safe, effective, and clinically relevant component of modern veterinary ophthalmic practice.

## Data Availability

No new data were created or analyzed in this study. Data sharing is not applicable to this article.

## Supplementary Materials

The following supporting information can be downloaded at: https://www.mdpi.com/article/10.3390/vetsci13050461/s1, Table S1: Main antiseptics used in veterinary ophthalmology, their mechanisms of action, antimicrobial spectrum, and clinical considerations.
