# Supplementary material for: Beyond Antibiotics: The Expanding Role of Non-Antibiotic Therapies in Veterinary Ophthalmology
Source: Vet Sci. 2026 May 9;13(5):461. doi: 10.3390/vetsci13050461 (PMC13211636; doi:10.3390/vetsci13050461)
Supplement: Supplementary file 1 [file vetsci-13-00461-s001.zip › vetsci-4242072-supplementary.pdf]

Table S1. Main antiseptics used in veterinary ophthalmology, including their mechanisms of action, antimicrobial spectrum, and key clinical considerations. Commercial formulations available for veterinary use, as well as human products commonly used off-label, are summarized. When applicable, veterinary products available in other countries and potentially accessible through importation are also indicated. OS: ocular surface.

Antiseptic	Mechanism of action	Antimicrobial spectrum	Indications	Comments	Available products (*veterinary, **human) in Europe	Available products (*veterinary, **human) in Unites States
**Povidone-iodine**	Denatures proteins, lipids, and nucleic acids	Gram-positive bacteria<br>Gram-negative bacteria<br>Fungi, viruses, protozoa, spores, biofilms	Perioperative antiseptic<br>Mild OS and adnexal disease	Concentration 0.2–1%<br>Irritant at higher concentrations	*Visiotears® (Sava Vet, IN)<br>**Minims® Povidone Iodine 5% (Bausch & Lomb, UK)<br>**Oftasteril® 5% (Brill Pharma, UK)<br>**Iodim® 0.6% (Angelini Pharma, IT)<br>**Refresh® Classic Lubricant Eye Drops 0.6% (Allergan, IRL)	**iVIZIA® Eyedrops and Gel 0.5% (Thea Farma inc, US)
**Polyhexanide (PHMB)**	Denatures membrane phospholipids	Gram-positive bacteria<br>Gram-negative bacteria<br>Fungi, protozoa, and biofilms	Perioperative antiseptic<br>Mild OS and adnexal disease	Effective against *Acanthamoeba* spp.<br>Concentration 0.02–0.08%	*Septostil® (Vetilea, ES)<br>**Ocudox® spray (Brill Pharma, UK)<br>**Akantior® (Faes Farma, ES)	Compounded prepatarations
**Hypochlorous acid**	Induces oxidative damage	Gram-positive bacteria<br>Gram-negative bacteria<br>Fungi, viruses, and biofilms	Perioperative antiseptic<br>Mild OS and adnexal disease	Concentration 0.01%	*Hypochlorine Eye Care® (JTPharma, ES)<br>**Ocudox® spray (Brill Pharma, UK)<br>**Hypochlorous Eyelid Cleansing Spray® (The eye doctor, UK)	*Vetericyn Plus® Antimicrobial Ophthalmic Gel (Innovacyn, USA)<br>*MicrocynAH Ophthalmic Gel® (Compana Pet Brands, USA)<br>*HICC Pet® Gentle Antimicrobial Pet Eye Rinse For Dogs (HICC Pet, USA)<br>**Noveha® antimicrobial eyelid cleanser (Noveha, USA)
**EDTA**	Chelates divalent cations (Ca, Zn), enhancing antibiotic efficacy	Adjuvant to antibiotics, improving penetration and limiting biofilm formation	Mild OS and adnexal disease<br>Chronic or persistent infections (as adjuvant)	Apply 5 minutes before other medications<br>Not recommended for >15 days<br>Combined with Polysorbate 80 and/or Tris	*Optican Limpiador de ojos® (Stangest, ES)<br>**Lacrifresh® Comfort Drops (AZIVOR, ES)<br>**ASTER® Trisoftal Wipes (VetNova, ES)<br>**Refresh Optive® Advanced (Allergan, IRL)	*Optixcare Eye Care® (CLC Medica, CA)<br>*LID 'N LASH VET® (I-MED Animal Health, CA)<br>**EDTA Compounded Ophthalmic® (Epicur, USA)<br>**IrisOphtho® Eye Wipes (DermaZoo™ Pharma, USA)

						**Avitears™ - Lubrication Eye Drops (Pinnacle Science, USA)
**Boric acid**	Alters microbial enzymatic metabolism	Mild antiseptic activity	Chronic ocular cleanser; reduces tear-staining syndrome<br>Mild OS and adnexal disease	No comments	*Ocryl® (Domes Pharma, FR)<br>*Siccostil Protect® (Vetilea, ES)<br>**Optrex® (Reckitt Benckiser Healthcare, UK)	*Lavatears® (Santgar SA, USA)<br>*Angels' Eyes® (H&C Animal Health, USA)<br>**GenTeal® Tears (Alcon Laboratories, USA)
**Hexamidine**	Disrupts microbial cell membrane	Gram-positive bacteria<br>Some Gram-negative bacteria<br>Protozoa	Mild OS and adnexal disease	Effective against *Acanthamoeba* spp. (less than PHMB)	*Septostil® (Vetilea, ES)	**Desomedine® 0.05% Eye Drops (Bausch and Lomb, USA)
**Ozone**	Induces oxidative damage	Gram-positive bacteria<br>Gram-negative bacteria<br>Fungi, viruses, and biofilms	Ocular surface cleanser, especially for corneal ulcers or infectious keratitis<br>Ozonated oils for periocular wounds or infections	Use at low concentrations (<5 ppm in water, <20 µg/mL in eye drops) to minimize risk of chemical keratitis	**Ozonest® (Laboratorios Esteve, ES)<br>**Oftasecur® (Offhealth, IT)	Not available.
